# iCHECK-DH: Guidelines and Checklist for the Reporting on Digital Health Implementations

**DOI:** 10.2196/46694

**Published:** 2023-05-10

**Authors:** Caroline Perrin Franck, Awa Babington-Ashaye, Damien Dietrich, Georges Bediang, Philippe Veltsos, Pramendra Prasad Gupta, Claudia Juech, Rigveda Kadam, Maxime Collin, Lucy Setian, Jordi Serrano Pons, S Yunkap Kwankam, Béatrice Garrette, Solenne Barbe, Cheick Oumar Bagayoko, Garrett Mehl, Christian Lovis, Antoine Geissbuhler

**Affiliations:** 1 Department of Radiology and Medical Informatics Faculty of Medicine University of Geneva Geneva Switzerland; 2 Geneva Digital Health Hub Geneva Switzerland; 3 Faculty of Medicine and Biomedical Sciences University of Yaoundé 1 Yaoundé Cameroon; 4 PATH Geneva Switzerland; 5 BP Koirala Institute of Health Sciences Dharan Nepal; 6 Government Innovation Bloomberg Philanthropies New York, NY United States; 7 Foundation for Innovative New Diagnostics Geneva Switzerland; 8 Fondation Pierre Fabre Castres France; 9 Novartis Foundation Basel Switzerland; 10 UniversalDoctor Barcelona Spain; 11 International Society for Telemedicine & eHealth Basel Switzerland; 12 Centre d’Innovation et de Santé Digitale DigiSanté-Mali Université des sciences, des techniques et des technologies de Bamako Bamako Mali; 13 Centre d’Expertise et de Recherche en Télémédecine et E-Santé Bamako Mali; 14 Department of Digital Health and Innovation World Health Organization Geneva Switzerland; 15 Division of Medical Information Sciences Geneva University Hospitals Geneva Switzerland

**Keywords:** implementation science, knowledge management, reporting standards, publishing standards, guideline, Digital Health Hub, reporting guideline, digital health implementation, health outcome

## Abstract

**Background:**

Implementation of digital health technologies has grown rapidly, but many remain limited to pilot studies due to challenges, such as a lack of evidence or barriers to implementation. Overcoming these challenges requires learning from previous implementations and systematically documenting implementation processes to better understand the real-world impact of a technology and identify effective strategies for future implementation.

**Objective:**

A group of global experts, facilitated by the Geneva Digital Health Hub, developed the Guidelines and Checklist for the Reporting on Digital Health Implementations (iCHECK-DH, pronounced “I checked”) to improve the completeness of reporting on digital health implementations.

**Methods:**

A guideline development group was convened to define key considerations and criteria for reporting on digital health implementations. To ensure the practicality and effectiveness of the checklist, it was pilot-tested by applying it to several real-world digital health implementations, and adjustments were made based on the feedback received. The guiding principle for the development of iCHECK-DH was to identify the minimum set of information needed to comprehensively define a digital health implementation, to support the identification of key factors for success and failure, and to enable others to replicate it in different settings.

**Results:**

The result was a 20-item checklist with detailed explanations and examples in this paper. The authors anticipate that widespread adoption will standardize the quality of reporting and, indirectly, improve implementation standards and best practices.

**Conclusions:**

Guidelines for reporting on digital health implementations are important to ensure the accuracy, completeness, and consistency of reported information. This allows for meaningful comparison and evaluation of results, transparency, and accountability and informs stakeholder decision-making. i-CHECK-DH facilitates standardization of the way information is collected and reported, improving systematic documentation and knowledge transfer that can lead to the development of more effective digital health interventions and better health outcomes.

## Introduction

### Background

In recent years, we have seen an exponential increase in the implementation of digital health technologies, as more and more health care organizations, providers, governments, and users recognize the benefits they can bring. From improving access to care and reducing costs to enhancing the patient experience and enabling more personalized care, the potential of digital health is vast and continues to grow.

A major challenge in digital health is that despite the rapid growth of digital health solutions, many of these implementations remain limited to pilot studies and fail to achieve widespread adoption. This phenomenon, known as “pilotitis” or “pilotitis syndrome,” refers to the tendency to focus on small, short-term pilot projects rather than implementing health technologies at scale.

There are several reasons digital health implementations may not progress beyond the pilot stage: The pilot does not provide sufficient evidence of effectiveness or value to justify a wider rollout [1], or there are challenges or barriers to implementation that are not addressed or resolved during the pilot phase, such as resistance from health care providers, a lack of stakeholder engagement, or policy-level barriers [2-4]. However, moving to widespread adoption of digital health technologies is critical to realizing their full potential. This means carefully assessing the evidence for the effectiveness and value of a technology, understanding and addressing the challenges or barriers to implementation, and developing strategies for scaling up successful pilots.

Overcoming implementation challenges in digital health requires learning from previous implementations, for example, by documenting implementation processes in a standardized format using reporting guidelines. Systematically learning from implementations enables a better understanding of the real-world impact of a technology, including its long-term effectiveness and unintended consequences. This can inform the development and implementation of future digital health implementations by identifying effective strategies and areas for improvement.

### Digital Health Implementations and Interventions

For this paper, intervention refers to the deliberate effort to change a situation or modify behavior, and implementation refers to the process of achieving the goal (how the intervention was carried out). Digital health is defined as the use of information and communication technologies for health. As with this definition, the scope is broad, and interventions can range from targeting an individual to strengthening components of health systems. Examples include tracking individual parameters, managing logistics for vaccine delivery, or conducting advanced data analytics for precision medicine. The World Health Organization's (WHO) Classification of Digital Health Interventions provides a good overview of the potential range of applications by organizing them into 4 umbrella groups based on the target primary user: (1) interventions for clients, (2) interventions for health care providers, (3) interventions for health system or resource managers, or (4) interventions for data services [5].

### Existing Guidelines

The value of standardized guidelines is well recognized. Various guidelines and tools exist for reporting scientific evidence, such as the Preferred Reporting of Systematic Reviews and Meta-Analyses (PRISMA) [6], the Consolidated Standards of Reporting Trials (CONSORT) for randomized controlled trials [7], the template for intervention description and replication (TIDieR) [8], the Strengthening the Reporting of Observational Studies in Epidemiology (STROBE) [9], the Standards for Reporting Qualitative Research (SRQR) [10], or the Case Report (CARE) statement and checklist [11].

In the field of digital health, the Consolidated Standards of Reporting Trials of Electronic and Mobile Health Applications and Online Telehealth (CONSORT-EHEALTH) [12] is an extension of the CONSORT statement that provides guidance on reporting trials of web-based interventions (eHealth) or mobile health (mHealth). It consists of 17 essential subitems. Notably, there are 4 subitems that relate to the important issue of attrition (not using) and use (engagement, dosage, adherence) of the intervention, which are often underestimated, even though they are critical factors in influencing the future impact of an intervention. There is further potential to use the checklist beyond web-based and mobile interventions and in settings where a lack of internet connectivity would not be a limiting factor. This will require further research into the specifics of such cases.

Finally, the mHealth Evidence Reporting and Assessment (mERA) checklist [13], developed by the WHO-led mHealth Technical Evidence Review Group (mTERG), aims to improve the completeness of reporting of mHealth interventions. The checklist seeks to identify “a minimum set of information needed to define what the mHealth intervention is (content), where it is implemented (context), and how it has been implemented (technical features) to support replicating the intervention” [13]. In its conclusions, mTERG clearly emphasized that the mERA checklist “is intended not only to assist authors in reporting mHealth research, but also to guide reviewers and policy makers in synthesising high-quality evidence, and finally to guide journal editors in critically assessing the transparency and completeness of reporting of mHealth studies” [13]. These guidelines are unique in that they are specific to 1 type of digital health intervention, but they also provide practical information about how to report technical details that go beyond simply reporting the design of a study and its scientific protocol. Lessons should then be learned to replicate the specific mHealth intervention described in the report.

Although both CONSORT-EHEALTH and mERA address reporting, the Guidelines and Checklist for the Reporting on Digital Health Implementations (iCHECK-DH, pronounced “I checked”) focus on the standardization of implementation reporting. CONSORT-EHEALTH is designed for reporting trials and provides a basis for improving transparency and consistency in the presentation of research on eHealth interventions, while mERA is tailored to mHealth interventions and provides a framework for assessing and reporting the evidence generated by these studies.

The literature review highlights the existence of various standards and guidelines for reporting scientific evidence and interventions; however, to the best of our knowledge, there are no guidelines supporting the reporting of digital health implementations and processes. Therefore, a global expert review panel was convened to fill this gap, resulting in the guidelines for the reporting of digital health implementations (iCHECK-DH). iCHECK-DH aims to improve our understanding of the intricacies of implementing digital health interventions in real-world settings by streamlining the documentation of implementation processes and contextual factors surrounding the use of digital health interventions.

This paper aims to describe the objectives, development process, and items of iCHECK-DH.

## Methodology

The methodology outlined by Moher et al [14] was used to develop iCHECK-DH.

To define the initial draft list of reporting items, we conducted a literature review that included searching multiple databases (PubMed, Google Scholar) for papers describing digital health implementations in different settings. Papers that focused on digital health technologies but did not discuss implementation were excluded. A set of 10 selected papers from different settings (eg, primary care, community care) were reviewed for common reporting elements as input for the first draft checklist. This initial draft checklist was developed by a team of 2 researchers and reviewed for completeness by a range of global stakeholders with different backgrounds and roles (researchers, implementers, technical experts). This resulted in a preliminary list of 27 reporting items.

**Figure 1:**
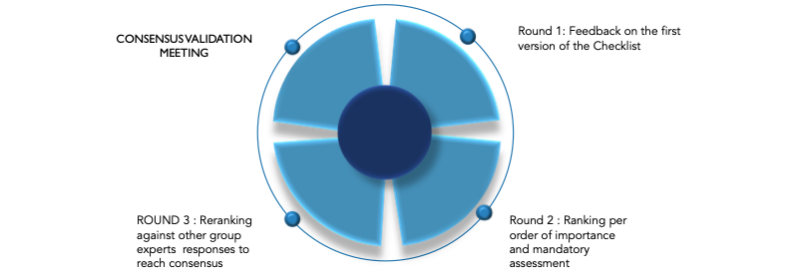
Steps in the Delphi process of the development of iCHECK-DH: Guidelines and Checklist for the Reporting on Digital Health Implementations.

A guideline development group of 18 experts was then established in September 2022 to support the development of the guidelines and checklist. The group includes experts from different areas of digital health, such as policy experts, funders, researchers, and implementation stakeholders. A 3-step modified Delphi method was used to review the checklist and reach a consensus on the items and whether they should be mandatory (see Figure 1).

In round 1, participants in the guideline development group reviewed the draft checklist and got an opportunity to add additional items. In round 2, the completed checklist was circulated to the experts, and they were asked to rate each item in terms of importance and whether it should be mandatory. Rankings were summarized using the median (IQR) and included in a repeat version of the questionnaire. The IQR is a measure of statistical dispersion and can be used as a measure of consensus among specialists, defined as a difference between the 75th and 25th percentiles or a range between upper and lower quartiles.

In round 3, the experts reranked their agreement with each statement, with the option to change their score in the light of the group response. Round 3 rankings were summarized and assessed for consensus using IQRs for continuous numerical scales and were accepted if the IQR was 2 or less. The result was a preliminary checklist, which was presented to the expert group in the consensus meeting.

In November 2022, the Geneva Digital Health Hub (gdhub) at the University of Geneva, initiator of the guidelines process, convened 2 different consensus expert meetings in Geneva and online. During these meetings, each item was discussed and some items were reorganized and regrouped, resulting in a final list of 20 items, the content and structure of which was agreed upon by all members of the guideline development group.

Selected items from the final checklist were tested in 2 workshops at international digital health conferences (Kathmandu, November 2022; Washington, December 2022). The full checklist was then applied to report on implementations (with different scales, contexts, and settings) to test its applicability and comprehensiveness. An implementation report for 3 of these was submitted for publication [15-17].

## Explanation and Elaboration of iCHECK-DH

In this section, the 20 selected items of the checklist (Multimedia Appendix 1) are described, including their rationale for inclusion, explanation, and examples to illustrate what is expected for each item.

The checklist consists of 7 sections: Title (item 1), Abstract (item 2), Introduction (items 3-5), Methods (items 6-14), Implementation (Results; items 15-18), Discussion (item 19), and General (item 20).

Items were classified as either mandatory (M) or nonmandatory (NM). Mandatory items were assessed as essential information to be provided by the authors of the intervention.

The format of the checklist may be used for reporting. However, authors can change the order of the items if they keep the items under the subheadings. Authors should report on all mandatory items, and if they do not report on a mandatory item, an explanation should be provided (eg, if there is no evaluation, provide a detailed explanation of the reasons).

### Item 1: Title (M)

Identify as an implementation report and describe the implementation in the title, keywords, or both.

#### Examples

- “Chatbot-Based Assessment of Employees’ Mental Health: Design Process and Pilot Implementation” [18]
- “Creation and Global Deployment of a Mobile, Application-Based Cognitive Simulator for Cardiac Surgical Procedures” [19]
- “The Journey to National Scale of Zanzibar’s Digitally Enabled Community Health Program: An Implementation Report” [15]

#### Explanation

Authors should choose a title that is concise yet accurately describes the purpose of the document as an implementation report to ensure maximum visibility and easy access. Strategic use of keywords in the title can make this document searchable in various publication databases for optimal reach and machine processing, without the need to explicitly mention “implementation.”

### Item 2: Abstract (M)

Provide a summary of the key elements of the implementation report, including a description of the implementation strategy and the intervention, defining the key elements of the implementation and health outcomes, and specify the key performance indicators (KPIs)/outputs.

#### Example

- “Background: Patient empowerment can be associated with better health outcomes, especially for chronic diseases. Concerto is a mobile application designed to promote patient empowerment in an in-patient setting. Methods: The application was designed and prototyped during a hackathon. It uses data from the hospital information system to provide key functionalities: a care plan, access to targeted medical information, practical information, information about the nursing team on duty, and a medical round preparation module. Following a feasibility study, funding was obtained, and the application developed using an agile methodology and deployed in 4 pilot divisions, using institution-owned iPads. Results: The project lasted for two years with effective implementation in the 4 pilot divisions, within budget. The induced workload on caregivers was a key challenge that warranted a change in our implementation strategy. The presence of a killer-function would have facilitated the deployment. Furthermore, our experience is in line with the well-accepted need for both a high-quality user-training and a good selection and engagement of super-users. By presenting Health Information System data directly to the patient, Concerto has highlighted them to be not fit-for-purpose and has triggered data curation initiatives. Finally, connecting the application to the HIS has promoted the usage of standards, both on the HIS and mobile applications side, that should facilitate future initiatives. Discussion: This implementation report presents a real-world example of implementing a patient-empowering mobile application in an in-patient setting of a University Hospital. One limitation of the study is the lack of definition of a Key Success Indicator” [16].

#### Explanation

The abstract provides the reader with a concise overview of the background information and progress toward the objectives. We recommend describing the main aspects of the research in the following order: background - objectives - methods - implementation (results) - conclusions - (optional: trial registration).

If applicable, include measurable KPIs and add keywords at the end of the abstract. In general, an abstract is around 150-300 words.

### Introduction Items 3-5

#### Item 3: Context (M)

Describe the geographical areas, organizations, target populations, and implementation context. Consider social, cultural, economic, political, health care, and organizational barriers; infrastructures; and facilitators that may influence implementation elsewhere. Explicitly highlight whether a national digital health strategy exists and whether implementation is aligned with the strategy.

Describe the stage of the implementation (developing or adapting solution/piloting and evidence generation/package and advocacy/acceleration/deploying/scaling up/hand over or complete).

##### Examples

- “Concerto is a mobile application aimed at promoting empowerment for hospitalized patients. This implementation report will focus on the initial pilot study” [16]. “Switzerland has a digital health strategy, which has a component that focuses on the promotion of mobile health, based on the mhealth recommendations” [20].
- “This study was a pilot implementation of a chatbot-based mental health assessment performed in a real-world workplace setting, based on a cross-sectional analysis. The sample comprised employees of an industrial plant in Sao Paulo, Brazil” [18].
- “This study reports the user-centered design and feasibility study of a chatbot to collect linked data about diet, physical activity, weight, obesity risk, living area, and social network to support research regarding individuals and social causes of obesity and overweight. Here, we describe the user-centered approach applied in the design and development of the chatbot. We also present a pilot study to test the chatbot’s feasibility” [21].

##### Explanation

Context refers to the specific geographic, social, economic, and political factors that may influence the success of the implementation. These factors include geographic location, such as a specific region, country, or urban/rural setting, and the social and economic context, including demographics, income levels, and cultural norms that may explain user adoption. It is also important to understand whether there is a national digital health strategy in place and how the implementation fits into this overarching strategy, as failure to do so may result in missed opportunities for adoption. In addition, authors should mention the stage that the implementation report describes, as different reports may be issued for different stages.

#### Item 4: Problem Statement (M)

Describe the health care or public health problem, challenge, or deficiency that the implementation aims to address. (If applicable, include a reference to the “health system challenge” of the WHO Classification of Digital Health Interventions [5] in the description.)

##### Examples

- “During the last decades, medicine has been moving from paternalistic approaches towards patient-centeredness and patient partnership and participation. Despite the high standard of healthcare in Switzerland, patients in hospital settings still face barriers to being fully involved in their own care and decision-making processes. This can lead to dissatisfaction with the care received, decreased trust in the healthcare system, and a lack of engagement in self-care and health management. Patient empowerment refers to a meta concept with no unique definition. It is however commonly accepted that empowered patients possess key capacities and resources to be able to participate in shared decision-making, manage their own health, and self-empower themselves. Patient empowerment can be associated with better outcomes, including mortality, especially for chronic diseases” [16].
- “Within hospital settings, a skills drills or emergency team may appoint a timekeeper in order to record clinical events in real time. However, within the home birth and other community-based settings, lone workers may also be faced with obstetric emergency, either alone or with little support. The unpredictability of such a scenario may be difficult to manage. As the need for clinical midwifery management becomes immediate, clinical record keeping may become retrospective and secondary to primary care” [22].

##### Explanation

The description of the health care or public health pain points, challenge, or deficiency that the intervention aims to address should be detailed and specific. This may include issues related to access to care, quality of care, patient outcomes, or health care delivery. It could also address public health challenges, such as disease prevention and control, health promotion, or population health management. It is important to clearly articulate the problems or deficiencies that the intervention aims to address to ensure that it is properly targeted and can effectively address the identified issues. Where appropriate, authors may wish to include a reference to the “health system challenge” of the WHO Classification of Digital Health Interventions in the description of this item [5].

#### Item 5: Similar Interventions (M)

Mention whether this implementation was inspired by another existing one. If so, what is the added value of your intervention, if any, compared to the initial one, and what, if anything, has been done differently?

##### Example

- “This health promotion program will be also based on successful and already tested health interventions for improving SRH of young people (including Cape Verdean youngsters), adapted to the needs and cultural-embedded values of the Cape Verdean young population, taking into account the perceptions of local health and educational professionals. This study presents an added value as a health promotion intervention with a comprehensive approach of well-being and quality of life across the life course. The framework of social prescribing and digital health literacy that supports this SRH intervention will allow social cohesion, throughout the cooperation between health and educational, from public or private sectors, including relevant stakeholders. A positive impact in social support perception is also expected, which is relevant from the perspective of sustainability. It is projected that the implementation and effectiveness assessment will embrace a pilot for national implementation, allowing to benefit several Cape Verdean’s local communities, health services, educational programs, and policymakers” [23].

##### Explanation

References to similar interventions can provide links between different projects, acting as a catalyst for synergies and thus optimizing the impact of interventions on the target populations or within a particular health system. An intervention could build on another pilot as a foundation, but be (1) an improvement, (2) a completely different initiative, or (3) a complement to the work of the base project. A critical point would be to demonstrate sufficient differentiation and strong added value of this specific intervention.

### Methods Items 6-14

#### Item 6: Aim and Objectives (M)

Describe the main objectives and the overall aim of the implementation. Describe how these will be measured using predefined primary and secondary outcome(s) and KPIs for this implementation and the expected intervention(s).

##### Examples

- “Our goal was to develop a system that will facilitate secure, trustable management, sharing, and aggregation of EHR data. Our patient-centric system allows patients to manage their own health records across multiple hospitals. The system will ensure patient privacy protection and guarantee security with respect to the requirements for health care data management, including the access control policy specified by the patient” [24].
- “Outcomes: Any positive or adverse health-based outcome and/or predictive indicators assessing pain and/or physical functioning/disability. Secondary outcomes: Any positive or adverse health-based outcome and/or predictive indicators assessing patient knowledge and understanding, self-efficacy, catastrophizing, and empowerment” [25].
- “The primary outcome was the change in insomnia symptom severity (measured by the Insomnia Severity Index) from baseline to postintervention. Secondary outcomes were sleep efficiency and nightly sleep duration (defined by sleep diary), global sleep quality (measured by the Pittsburgh Sleep Quality Index), depressive symptom severity (measured by the Edinburgh Postnatal Depression Scale), and anxiety symptom severity (measured by the Generalized Anxiety Disorder Scale-7)” [26].

##### Explanation

A detailed description of the objectives and the overall aim of the implementation gives the reader a clear idea of what the implementation is about. Authors should also clarify the difference between implementation objectives (the process; eg, with KPIs) and intervention objectives (the effectiveness), with outcome indicators, where appropriate. For example, authors might consider indicators or proxies that measure direct health outcomes (eg, hemoglobin A1C [HbA1c] for patients with diabetes), KPIs (eg, number of users, number of users properly trained, user satisfaction), and an indicator that assesses a particular process (eg, administrative time for patient admission). If no evaluation of objectives or outcomes has been carried out, it is important to explain the reasons for this decision.

#### Item 7: Blueprint Summary (M)

Describe the design and key features of the intervention and key points of the implementation strategy and roadmap.

##### Examples

- “It (Concerto) was conceptualized and prototyped during a hackathon in 2015 by a multi-disciplinary team including healthcare and IT professionals as well as one patient. Building on the hackathon prototype and after a feasibility study, the Geneva University Hospitals launched a project aiming at developing a fully functional mobile application to be deployed on institution-owned iPads in 4 divisions: oncology, neurorehabilitation, orthopaedics, and paediatrics, and assessing its effectiveness. Following this pilot study, the mobile application was further refined and deployed institution-wide in a Bring-Your-Own-Device (BYOD) approach” [16].
- “…we developed pain SELfMAnagement (SELMA) as a text-based health care chatbot (TBHC) for the self-management of chronic pain. A TBHC is a conversational agent that supports health professionals in the delivery of evidence-based interventions in a ubiquitous and fully automated fashion with simple text-based messages and media objects (eg, videos, podcasts). A TBHC aims to deliver the treatment and to increase working alliance by communicating therapeutic goals and tasks in an empathetic way. Against this background, we here describe the design and implementation of SELMA, an 8-week smartphone-based TBHC intervention for self-management of pain by patients with ongoing or cyclic pain, and present findings from a pilot randomized controlled trial, in which effectiveness, acceptance, and adherence were evaluated” [27].

##### Explanation

When describing the implementation strategy and the main features of the intervention, authors should bear in mind that intervention refers to the deliberate and intentional effort to change a situation or behavior and implementation refers to the process of achieving the goal (how the intervention is carried out). Outline the implementation strategy with key milestones. This will provide a more complete understanding of the implementation process and any deviations from the original plan. If possible, authors can include “Digital Health Interventions” and “System Categories” from the WHO Classification of Digital Health Interventions [5].

#### Item 8: Technical Design (M)

Specify reasons for developing or choosing this tool. Does it combine several tools? Provide a brief description of the tools (functionality and architecture) and how it fits into the health enterprise architecture and investment roadmap (if applicable). Indicate whether the solution is based on an existing solution or has been developed or purchased specifically for this intervention.

Describe the type of technology used (eg, artificial intelligence [AI] applications) and the license of the technology (open source, free, commercial, intellectual property [IP] ownership, etc) and include code documentation (if available), a link to the application, and a link to wiki or the project website.

##### Examples

- “The Jamii ni Afya mobile app is built on the Community Health Toolkit (CHT), an open source global goods platform developed to support community health workers globally. This platform was selected by the Zanzibar government due to the following reasons: it is open-source and uses well-known components and frameworks; it has a growing community that can be leveraged for support; it can be hosted in a local datacenter; the skills required to configure health worker tools are found among Ministry ICT staff and easily available in the local market. In addition, CHT runs on low-end Android smartphones and has offline functionality, which is critical in Zanzibar where network connectivity is not guaranteed”[15].
- “The application was developed in a web-based, responsive, coding language, encapsulated for iOS and deployed on institution-owned iPads using a mobile device management. The key arguments for internal development over acquisition of a commercial solution were that (1) most of the development work was about interfacing with the Hospital Health Information System, (2) no mature commercial solution was available at that time, to our knowledge” [16].
- “The Java programming language was used to develop a native app focusing on the Android platform, in which personal and medical data are maintained using the SQLite database. The first version of the app was con-structed and presented to the same nephrologist still in 2015. This aimed to make sure that all specified requirements were incorporated into the app” [28].

##### Explanation

Technical design explains the reasoning and process behind the implementation of a specific functionality. Understanding the lessons learned or failures (assessing the actual technical design against the original intent) can help improve digital health development standards and address common technical issues encountered in implementations.

#### Item 9: Target (M)

The target refers to the focus or recipient of the intervention. It is the individual, group, system, or problem that the intervention aims to change or improve. Describe the characteristics of the targeted “site(s)” (locations, staff, resources, etc) for implementation and any eligibility criteria, as well as the population targeted by the intervention and any eligibility criteria.

##### Examples

- “Concerto is a mobile application designed to promote empowerment for patients still hospitalized or released from the hospital and ease the support provided by their caregivers, in Switzerland” [16].
- “This app development is conducted as it is essential for fulfilling the need to increase midwives’ competencies integrated with their services based on mobile health that can facilitate midwives to develop themselves according to their profession. ‘’ subjects included midwife participants in West Java Province, Indonesia” [29].
- “Jamii ni Afya leverages government guidelines and global best practices to guide CHWs using digital technology in delivering high quality, health education and counseling services in maternal and child health, nutrition, water, sanitation and hygiene (WASH) and early childhood development” [15].

##### Explanation

The targets may include the sites, the staff, or other resources needed for the implementation, as considered by the authors.

It is important to accurately describe the targets of the intervention, including those added during the implementation process, in order to provide a full understanding of the scope and objectives of the intervention. In some cases, the rationale for the choice of targets may be inadequately explained or overlooked, as the authors may assume that it is obvious from the context. It is important to clearly describe the eligibility criteria for all targets of the intervention to provide a thorough understanding of the target selection process. An exhaustive description will facilitate not only the assessment of relevance but also, where appropriate, the replication of the intervention by others.

#### Item 10: Data (M)

Describe the data governance, including the life cycle (collection, processing, storage, modification, sharing, suppression); data ownership (mention whether patients actually have access to the data); data protection measures; confidential use of routine data; expected level of data integration; data for research; cross-border data agreement, if any; the applicable legal framework; and how the project complies with it. Describe data consent: Has patient consent been obtained? Describe the approach to data protection and cybersecurity (eg, security by design, privacy by design) and where the data are hosted (eg, in-country, cloud-based, hybrid model). If applicable, describe the government’s data policy.

##### Examples

- “Concerto was mainly ‘read-only’ for personal health data present in the HIS and for unsensitive, unpersonal information. The information patients accessed from the HIS were part of its medical record. According to the Swiss law, every patient owns data from his medical record, except for personal notes of healthcare professionals which were out of the scope of concerto. Accordingly, concerto facilitated the access to data already owned by the patients. The only personal information entered in concerto was questions patients may have before interaction with caregivers. This information was stored locally on the iPad. As iPads were erased and reinitialised between patients, this information was systematically deleted. Secure login information based on the patient ID number and an SMS challenge also protected from unwanted access to sensitive personal information. Overall, the project was compliant with the Swiss law for data protection” [16].
- “The program consists of a clinic computer that hosts the local database of patient names and their phone numbers. A secure server, hosted by Sawubona Health, synchronizes with the local database. Each week, the server uses PHP scripts (commonly used for web development) to automatically generate the messages and pass them and the corresponding phone numbers to a BulkSMS service provider (Celerity Systems LTD, South Africa) for distribution” [30].

##### Explanation

Effective data strategies are essential for the successful implementation of digital health, while complying with legal frameworks on data ownership and privacy. Transparency of data used for decision-making is critical to facilitate evaluation of interventions. Ethics committees may require reporting of the detailed data strategy.

#### Item 11: Interoperability (M)

Describe the interfaces (what other systems does the tool connect to) and the standards that were used (which specific ones and rationale of choice; eg, semantic ontologies, such as the International Classification of Diseases [ICD], Systemized Nomenclature of Medicine – Clinical Terms [SNOMED CT], Logical Observation Identifiers, Names and Codes [LOINC], or technical standards, such as Health Level Seven Fast Healthcare Interoperability Resources [HL7 FHIR]).

##### Examples

- “The initial version of the application connected directly to the custom-made Geneva University Hospitals Health Information System (HIS) using proprietary interfaces. Further versions of the application have used industry standards such HL7/FHIR to connect to REST/APIs on the hospital side. This new architecture has required significant evolutions both on the application and the HIS side” [16].
- “NextGen Connect Integration Engine is also a cross-platform engine allowing the bidirectional sending of messages in many supported standards (eg, HL7 V2, HL7 V3, HL7 Fast Healthcare Interoperability Resources, DICOM) between systems and applications. Semantic interoperability provides interoperability at the highest level, which is the ability of two or more systems or elements to exchange information and to use the information that has been exchanged” [31].

##### Explanation

At the national level, the implementation of harmonized interoperable systems is essential to ensure sustainability and cost-effectiveness, as well as to optimize coordination between stakeholders. Interoperability can be achieved at different levels (technical, syntactic, semantic, organizational, or even legal). It is recommended to fully describe all standards and to mention whether interoperability policies have been applied (in an organizational or national context).

#### Item 12: Participating Entities (M)

Describe the following:

Implementing organization(s): type of organization(s), mission, leadership, vision, etc.

Government involvement: Describe whether the government was involved in the implementation, at what level, and at what stage(s).

Partners: Describe all partners (organizations) and their role in the implementation.

Funders: List all actors and stakeholders who have funded or invested in the development of the implementation (if different from the implementation, eg, using an existing digital health intervention). Indicate their level of involvement in terms of funding.

Mention which entity will own the final product and intellectual property after the implementation phase.

##### Examples

- “mTrac is a government initiative that originated as a pilot project within a Millennium Villages Project and Foundation for Innovative New Diagnostics (FIND). It was then handed over to the Government of Uganda for launch and scale up in December 2011. The Ministry of Health (MoH) fully owns and operates mTrac and it began to roll it out in four phases, each covering approximately twenty-eight districts.With financial support primarily from the UK Department for International Development (DFiD), this is done in three key ways. Firstly, via SMS, in order to transmit weekly surveillance reports (i.e. information on disease outbreaks and stocks of anti-malarials) from health facilities to the MoH and District Health Offices (DHOs). The MoH receives technical support from UNICEF and WHO, as well as financing from DfID, but mTrac is formally governed via a government-led Steering Committee chaired by the National Medical Stores, as well as via a dedicated eHealth Technical Working Group (TWG)” [32].
- “The feasibility study and initial concept were self-funded by the eHealth and Telemedicine Division of the Geneva University Hospitals and included salaries for a junior developer and a senior project manager. The project pilot was then funded by the Fondation Privée des HUG and included the aforementioned salaries as well as necessary materials (in particular iPads, covers and software licenses). No direct state-funding was provided at that stage of the project” [16].

##### Explanation

Identify all the different stakeholders involved in implementation. Partnerships can be key to successfully initiating and scaling up digital health tools. Therefore, understanding the respective roles of each participating entity will facilitate a clear understanding of the strategy and interactions.

#### Item 13: Budget Planning (M)

Describe the planned budget for implementation (include costs such as change management, user training, project management, technology pricing, total cost of ownership). If possible, include actual costs, otherwise describe the range or percentage of the total budget. Indicate the time frame covered by the budget. Describe the budget for the intervention (eg, development, purchase or adaptation of a free tool); if possible, include actual costs, otherwise describe them as a percentage of the total budget.

##### Examples

- “The cost of Corrie was estimated to be $229 per month per patient for a 1-year use term ($2750 per year). Based on this cost estimate, the use of the DHI leads to a cost-savings of $7274 per patient compared with standard of care alone (ie, $10,024 − $2750 = $7274). The $2750 figure is composed of a: (1) Bluetooth blood pressure monitor (∼$40); (2) refurbished smartwatch (∼$250); (3) medication pillbox (∼$10); (4) tote bag (∼$12.50); (5) printed instructions (∼$5); and (6) clinical support for onboarding and maintenance of platform by engineering inclusive of server storage fees and helpline access per user (∼$2432 annually)” [33].
- “This project was estimated at CHF 60, 000: CHF 8,000 for the adaptation of the Mediboard software; CHF 33,000 for investments (intranet network, computer equipment - servers, computers, switches, etc.), communication and marketing of the project; CHF 14,000 for operations (project management, change management, management of steering and working meetings - with Maternity user group); CHF 2,000 for training and support of users; and finally CHF 3,000 for the evaluation of the solution by an external evaluator of project” [17].

##### Explanation

A comprehensive budget plan with detailed cost estimates for both the implementation and the intervention is important for conducting future economic evaluations and for estimating future funding needs; budget planning helps to optimize resource allocation decisions, which can influence the effectiveness of digital interventions. One dimension of long-term ownership that should be considered is whether the tool has a “technical owner” who can provide technical support and own the functionality of the tool in the long term before it is adopted [34]. The costs associated with such resources need to be properly assessed. Where possible, authors should include real costs.

#### Item 14: Sustainability (M)

Describe the business model, including the sustainability model (financial, environmental, etc). If possible, relate outcomes to costs to assess sustainability. Describe long-term exit strategies and all dimensions considered to sustain the project after the end of the funding period. If applicable, describe the potential institutionalization of the project.

##### Examples

- “The University of Pittsburgh team provided all capital expenditures for the wellness center’s construction, as well as telehealth equipment, personnel recruiting and training, power and water infrastructure, and operating expenses for the first two years. The model was designed to be self-sustaining after two years of operation by 1) reimbursing services through government programs such as health insurance and 2) returns from the agribusiness enterprise that provide income and employment to the local community and generate enough economic value to support the Tuver Project. At the end of Year 1, all healthcare services, including specialty and super-specialty teleconsultations and basic laboratory tests at the wellness center were available at no cost. The project provided medicines free of charge to those with incomes below the poverty line and at discounted rates to all other patients. The program offered outreach services and menstrual hygiene kits at no cost to community members. The Common Services Center provided services at nominal prices predetermined by the relevant governmental authority” [35].
- “To ensure the sustainability of this project, several actions have been taken. These include: the alignment of the project’s objectives with those of YCH and its implementation with the agreement of the Director of YCH; the commitment of the Director to fund one third of the budget; the establishment of a steering committee including all YCH stakeholders, the integration of the YCH Information Technologies manager as deputy project manager, the implementation of a user group to assist the project group in the design and the implementation of the project, the designation of local champions (in the maternity) to provide leadership in the use of the CIS and finally, the official launch (supported by a document signed by the Director) of the beginning of the use of this CIS in the maternity” [17].

##### Explanation

Sustainable financing is critical to scaling up digital health interventions, but it is also often perceived as 1 of the most challenging parts of the process. Understanding whether the benefits of digital health services are balanced with their impact on the planet is important to ensure the continued advancement of the technology, while promoting sustainability. Authors should explain the sustainability plan (financial, environmental, etc) for implementation. If applicable, describe long-term exit strategies and all dimensions considered to sustain the project after funding ends.

One approach recommended by National Health Service (NHS) England to make digital health solutions sustainable in health systems was “to reject the traditional linear model of the innovation process in favour of an interactive model where implementation is not an afterthought but a primary focus of co-design efforts” and to move away from a focus on the technology itself to a focus on how digital technologies will be integrated and used in services in order to understand the context, environment, and constraints of the people who will use them and the target populations who will benefit from them [36].

### Implementation (Results) Items 15-18

#### Item 15: Coverage (M)

Describe whether the coverage of implementation is international, national, regional, or at the level of, for example, municipalities. If coverage is subnational, describe the regions. Provide information about the relative importance of the coverage (eg, percentage of the eligible population covered).

##### Examples

- “The Geneva University Hospitals, a 2000-beds Swiss teaching hospital, launched a project to develop a fully functional mobile application to be deployed on institution-owned iPads in 4 divisions: oncology, neurorehabilitation, orthopaedics, and paediatrics. Following this pilot study, the mobile application was refined and deployed institution-wide following a Bring-Your-Own-Device (BYOD) approach. Concerto was then further extended to encompass the ambulatory setting and facilitate the transition from hospital care to home care” [16].
- “This study took place at Webuye County Hospital, a rural hospital in Bungoma County with a catchment population of 500,000 people. It is estimated that approximately 44% of the population are children, 15 years. The hospital TB clinic cares for 200 patients with active TB annually. More than 1500 children are seen monthly in the pediatric outpatient clinics (nutrition, maternal child health, acute care)” [37].

##### Explanation

When describing the coverage of the implementation, authors might consider comparing the planned coverage (both geographical locations and population) with the actual results. Any discrepancies observed should be mentioned, as well as suggestions for better understanding the reasons for the differences.

#### Item 16: Outcomes (M)

Describe the primary and other outcomes of the implementation. Detail the actual outcomes, using the predefined outcome measures (if applicable).

##### Example

- “The FORA device, when used as an intervention within standard care as a control, had a moderate to large between-group effect on medication adherence 1 month posttest (d=0.77), 2 month posttest (d=0.88), and continued to stay just as effective, if not become even more so, at the 3-month follow-up (d=1.02)” [38].

##### Explanation

Authors should comprehensively describe the different levels of outcomes of the implementation and then evaluate the achievement of these against the initial strategy. This will help better assess the effectiveness of the project.

#### Item 17: Lessons Learned (M)

Describe any lessons learned from the implementation experience that could be used to improve future outcomes. This could include, but is not limited to, success factors, implementation challenges, or budget considerations.

Success factors: Describe factors that positively influenced the implementation (eg, involvement of key stakeholders). In addition, describe contextual factors that may have positively influenced the results (eg, new legislation that facilitated adoption).

Challenges to implementation: Describe challenges (process related, such as resistance to change, but also technical). Include contextual factors that may have affected the achievement of outcomes, such as an unexpected change of government or “opposing key players” who, despite potential participation, may hinder implementation (eg, software companies managing regional digital health may act as barriers to innovation).

Budget: Describe whether the implementation budget was met, and if not, why not. In addition, detail the expected operational costs (eg, licensing, maintenance, human resources, updates to in-house developments) to estimate the total cost of ownership. Include actual costs, otherwise describe them as a percentage of the total budget.

What recommendations can be drawn from the lessons learned?

##### Examples

- “Lessons learned, presented in the results section, are summarized in Table 1. The generalisability of our findings is low as they constitute the report of one implementation and may obviously vary in a different implementation context, such as another category of hospital, another healthcare system or another cultural context. ‘Overall, the order of magnitude of the project costs was comprised between 150k and 200k CHF, from which 25% was used for materials’” [16].

- “Related to the implementation of the NAA in practice, some unforeseen challenges arose that needed to be solved. For example, three healthcare workers were not able to attend the 2-day NAA training and were therefore trained individually at their healthcare facility. Also, of the in total 15 tablets five broke down, of which three could not be replaced and some remaining ‘bugs’ in the application were discovered. Healthcare workers did not always express their true opinions or would not call the technical support team when problems occurred. There was no script available in the implementation plan to guide responses to unexpected changes making it difficult to solve problems and ensure sustained use of the programme. Based on the lessons learned during the process of developing and implementing the NAA we recommend future programme developers to (1) engage the community and listen to their insights, (2), focus on clear programme goals and the desired change, (3), consult or involve a behaviour change specialist, and (4), anticipate potential problems in unexpected circumstances” [39].

**Table 1:** Main lessons learned and associated perceived relevance [16].

Lessons learned	Perceived relevance
Minimize or, if possible reduce the workload of caregivers.	5/5
Plan protected time to train end-users.	4/5
Communicate regularly to keep caregivers engaged.	4/5
Select convinced and influential superuser.	3/5
Wait for a killer function to implement the application.	5/5
Maturity of HIS in terms of interoperability standards facilitates implementation	5/5

##### Explanation

Failure to evaluate the different stages of development may prevent the understanding of key lessons that could support greater effectiveness and adoption of these initiatives. Authors should aim to formulate any recommendations that could serve as guidance for more effective implementation of digital health tools/systems.

#### Item 18: Unintended Consequences (NM)

Describe any unintended consequences (positive or negative), harms, or negative side effects (if any).

##### Examples

- “Our strategy to use institution-owned iPads has brought important additional workload on care teams as they were in charge to manage iPad fleets in their division. There was also positive consequence for Concerto which was able to be exposed in the Information systems” [16].
- “During this project and after implementing some approaches for change management (setting up a user group, identifying local champions, training of users, implementing news processes based on the procedure manual, setting up user support), we experienced an unintended or unexpected event which was staff reluctance to use the system after implementation due to lack of financial motivation. This situation required the project management team to put in place a special documentation gratification (per diem introduced to encourage staff to enter patient information into the system)” [17].

##### Explanation

During the implementation and intervention phases, unexpected events may occur that could potentially hinder implementation or, on the contrary, increase its impact. It is therefore important to identify any unintended consequences. This important assessment can lead to the revision or creation of standards.

#### Item 19: Discussion (M)

Provide a summary of the conclusions and future implications.

##### Examples

- “As in reaction to one of the main lessons learned, a Bring-Your-Own-Device version of the application was developed. With this version, every patient was able to use the application on its personal device (computer, tablet, or smartphone). This was done to limit the workload on caregivers and improve the adoption rate. New functionalities, including the possibility for patients to choose his meal, were also developed to answer unmet needs for both end-users and stakeholders impacted by the implementation of the application (i.e.: caregivers). A dedicated implementation report describing this project phase shall be submitted soon” [16].
- “The results from this study are added evidence that chronic disease risk reduction is achievable through a variety of modalities, including digital-based programs with human coaching. With the added advantage of accessibility and scalability, digital programs with human coaching should be an important part of the comprehensive health improvement solution for chronic disease risk reduction for older adults. This study demonstrated that older adults who agreed to participate in this program were able to engage meaningfully and gain important health and wellness benefits during a relatively short time frame” [40].

##### Explanation

Authors should use this summary section to help readers understand the main conclusions of the report. This section should also help the evaluators quickly understand the stakes and various future implications of the implementation.

#### Item 20: General (NM)

If applicable, include statements on regulatory approvals (eg, as appropriate, ethical approval, governance approval), trial or study registration (availability of protocol), and conflicts of interest. For implementation reports with a research component, ethical approval or a waiver from an appropriate ethics committee is required. For those without a research component, ethical considerations may still be relevant but do not necessarily require approval or a waiver. Authors may consult Eccles et al [41] for further guidance on ethical considerations in their specific context.

##### Examples

- “We conducted a randomized controlled pilot trial to assess the SELMA intervention. The clinical trial was approved by the Cantonal Ethics Committee of Zurich (KEK-ZH study protocol identifier Nr. 2017-02136) and was registered at the Swiss National Clinical Trial Portal (SNCTP000002712) and the WHO-accredited German Clinical Trials Register (DRKS00017147). Data protection requirements were fulfilled according to the KEK-ZH” [27].
- “A randomized control trial assessing the effectiveness of the Concerto mobile application on a patient situation awareness score has been designed and should be conducted soon. It will allow for a better evaluation of the cost-effectiveness of such project. Overall, data on the effectiveness of eHealth projects are often lacking, and the creation of the JMIR implementation reports is aiming to fill that gap” [16].

## Conclusion

### Key Messages

- iCHECK-DH aims to improve the reporting of digital health implementation knowledge and facilitate the identification of key success and failure factors.
- The checklist identifies a minimum set of information needed to comprehensively describe a digital health implementation (context, intervention, and implementation processes).
- These guidelines are intended to bridge the gap between implementation research and practice by promoting the documentation and dissemination of the perspectives and experiences of those involved in implementing digital health interventions and to improve the quality of digital health implementation standards and best practices.

Guidelines for reporting digital health implementations and processes are important for several reasons. First, they help ensure the accuracy and completeness of the information reported about an implementation, which is critical because the results of the implementation may be used to make decisions about the subsequent use of digital health technologies. Gaps in reporting can lead to these decisions being based on incomplete or inaccurate information. Second, guidelines help ensure that the information reported is consistent and comparable across different implementations. This allows for a meaningful comparison and evaluation of the results of different implementations. Finally, guidelines can help ensure the transparency and understandability of reported information, thus enabling stakeholders, such as health care providers, patients, and policy makers, to make informed decisions about the use of digital health technologies.

Guidelines for reporting on digital health implementations are needed to standardize the way information is collected and reported. This will help improve the systematic documentation and transfer of knowledge about digital health implementations, enabling lessons learned in one context to be applied in another. The use of these guidelines will also support the development of a body of evidence on digital health implementations that can be used to inform the effectiveness of digital health interventions and inform decision-making by researchers, practitioners, and policy makers.

It is important to note that iCHECK-DH is likely to evolve over time to reflect changing ecosystems as new technologies and implementation strategies will emerge. For example, they may need to be updated to address emerging issues or new types of digital health interventions. In addition, these guidelines will be applied to the Implementation Report section of the *JMIR Journal of Medical Informatics* [42]. Feedback from the application of these guidelines to a wider range of implementations may lead to modifications.

By following these guidelines, researchers and practitioners can ensure that their implementation reports are complete, accurate, and consistent, thereby facilitating understanding and learning from their work. This can ultimately lead to the development of more effective digital health interventions and better health outcomes for the populations served. In addition, the use of these guidelines can help promote transparency and accountability in digital health, allowing stakeholders to better understand the successes and challenges of different implementations.

## Appendix

Multimedia Appendix 1 Checklist of iCHECK-DH guidelines. iCHECK-DH: Guidelines and Checklist for the Reporting on Digital Health Implementations.

## Abbreviations

AI: Artificial Intelligence
CONSORT: Consolidated Standards of Reporting Trials
CONSORT-EHEALTH: Consolidated Standards of Reporting Trials of Electronic and Mobile Health Applications and Online Telehealth
gdhub: Geneva Digital Health Hub
HIS: Health Information System
HL7 FHIR: Health Level Seven Fast Healthcare Interoperability Resources
iCHECK-DH: Guidelines and Checklist for the Reporting on Digital Health Implementations
ICD: International Classification of Diseases
IP: intellectual property
KPI: key performance indicator
mERA: mHealth Evidence Reporting and Assessment
mHealth: mobile health
mTERG: mHealth Technical Evidence Review Group
NHS: National Health Service
WHO: World Health Organization
