# Correction: iCHECK-DH: Guidelines and Checklist for the Reporting on Digital Health Implementations

**DOI:** 10.2196/49027

**Published:** 2023-05-18

**Authors:** Caroline Perrin Franck, Awa Babington-Ashaye, Damien Dietrich, Georges Bediang, Philippe Veltsos, Pramendra Prasad Gupta, Claudia Juech, Rigveda Kadam, Maxime Collin, Lucy Setian, Jordi Serrano Pons, S Yunkap Kwankam, Béatrice Garrette, Solenne Barbe, Cheick Oumar Bagayoko, Garrett Mehl, Christian Lovis, Antoine Geissbuhler

**Affiliations:** 1 Department of Radiology and Medical Informatics Faculty of Medicine University of Geneva Geneva Switzerland; 2 Geneva Digital Health Hub Geneva Switzerland; 3 Faculty of Medicine and Biomedical Sciences University of Yaoundé 1 Yaoundé Cameroon; 4 PATH Geneva Switzerland; 5 BP Koirala Institute of Health Sciences Dharan Nepal; 6 Government Innovation Bloomberg Philanthropies New York, NY United States; 7 Foundation for Innovative New Diagnostics Geneva Switzerland; 8 Fondation Pierre Fabre Castres France; 9 Novartis Foundation Basel Switzerland; 10 UniversalDoctor Barcelona Spain; 11 International Society for Telemedicine & eHealth Basel Switzerland; 12 Centre d’Innovation et de Santé Digitale DigiSanté-Mali Université des sciences, des techniques et des technologies de Bamako Bamako Mali; 13 Centre d’Expertise et de Recherche en Télémédecine et E-Santé Bamako Mali; 14 Department of Digital Health and Innovation World Health Organization Geneva Switzerland; 15 Division of Medical Information Sciences Geneva University Hospitals Geneva Switzerland

In “iCHECK-DH: Guidelines and Checklist for the Reporting on Digital Health Implementations” (J Med Internet Res 2023;25:e46694), the authors noted one error.

The degree list for author Christian Lovis was previously reported as:

*MD, PhD*

And has now been changed to:

*MPH, MD*

The correction will appear in the online version of the paper on the JMIR Publications website on May 18, 2023 together with the publication of this correction notice. Because this was made after submission to PubMed, PubMed Central, and other full-text repositories, the corrected article has also been resubmitted to those repositories.

